# Photothermal-Controlled Release of IL-4 in IL-4/PDA-Immobilized Black Titanium Dioxide (TiO_2_) Nanotubes Surface to Enhance Osseointegration: An In Vivo Study

**DOI:** 10.3390/ma15175962

**Published:** 2022-08-29

**Authors:** Bo Chen, Yu Liang, Yunjia Song, Yunkai Liang, Jian Jiao, Hong Bai, Ying Li

**Affiliations:** 1 School of Dentistry, Tianjin Medical University, Tianjin 300070, China; 2 Tianjin Key Laboratory of Cellular and Molecular Immunology and Key Laboratory of the Educational Ministry of China, Department of Immunology, School of Basic Medical Sciences, Tianjin Medical University, Tianjin 300070, China

**Keywords:** photothermal, controlled release, IL-4, PDA, TiO_2_ nanotubes, osseointegration, near-infrared laser, titanium

## Abstract

Host immune response has gradually been accepted as a critical factor in achieving successful implant osseointegration. The aim of this study is to create a favorable immune microenvironment by the dominant release of IL-4 during the initial few days after implant insertion to mitigate early inflammatory reactions and facilitate osseointegration. Herein, the B-TNT/PDA/IL-4 substrate was established by immobilizing an interleukin-4 (IL-4)/polydopamine (PDA) coating on a black TiO_2_ nanotube (B-TNT) surface, achieving on-demand IL-4 release under near infrared (NIR) irradiation. Gene Ontology (GO) enrichment analyses based on high-throughput DNA microarray data revealed that IL-4 addition inhibited osteoclast differentiation and function. Animal experiment results suggested that the B-TNT/PDA/IL-4+Laser substrate induced the least inflammatory, tartrate-resistant acid phosphatase, inducible nitric oxide synthase and the most CD163 positive cells, compared to the Ti group at 7 days post-implantation. In addition, 28 days post-implantation, micro-computed tomography results showed the highest bone volume/total volume, trabecular thickness, trabecular number and the lowest trabecular separation, while Hematoxylin-eosin and Masson-trichrome staining revealed the largest amount of new bone formation for the B-TNT/PDA/IL-4+Laser group. This study revealed the osteoimmunoregulatory function of the novel B-TNT/PDA/IL-4 surface by photothermal release of IL-4 at an early period post-implantation, thus paving a new way for dental implant surface modification.

## 1. Introduction

Titanium (Ti) and Ti-based alloys have been widely used for dental implants due to their outstanding biocompatibility, mechanical strength and corrosion resistance properties [1,2]. However, in undesirable general conditions, such as osteoporosis, uncontrolled diabetes mellitus (DM) and cancer, clinical effects may be adversely influenced by reduced mineralized tissue amount, a compromised bone-wound healing process and poor systemic status [3]. In such cases, surface modification is of great importance to promote osteogenesis at the interface between dental implant and bone tissue [4]. 

Among the various methods of surface modification, titanium dioxide (TiO_2_) nanotubes (TNT) have attracted extensive attention due to their ideal properties including promoting rapid osseointegration and serving as drug release platforms [5,6]. A series of studies were performed on TNT surfaces and have proved their satisfactory osteogenic effects [7,8,9]. Moreover, we further reported that after combination of TNT with hydroxyapatite (HA) and human bone morphogenetic protein-2 (hBMP-2), the nanotopography could play a synergistic role with additional bioactive agents in promoting osteoblast adhesion, proliferation, differentiation and osseointegration [10,11]. In addition to their osteogenic effect, TNTs could also regulate the immune system by attenuation of macrophage inflammation and inhibit osteoclast activity, so as to improve osteogenesis and bone integration [12,13,14], implying a close relationship between the immune system and bone system. However, the underlying mechanism awaits further study.

Recently, osteoimmunomodulation (OIM) has evoked more and more attention in unravelling the sophisticated bone-implant healing process, which consists of initial hematoma formation, instant immunomodulation, angiogenesis and subsequent osteogenesis [15,16]. It has been reported that biomaterial implantation could immediately stimulate the innate immune system and enter the early stage of acute inflammation, which serve as the first step of tissue repair [17]. Inflammatory macrophages secrete many cytokines during the acute inflammatory phase to promote the osteogenic differentiation of mesenchymal stem cells [18]. Although initial acute inflammatory reaction plays an extremely important role in bone healing [19,20], long-term existence of inflammation may lead to chronic inflammation or fiber encapsulation and enhance the vitality of osteoclasts, which compromise complete osseointegration [16,18,21]. During the normal healing process, the inflammatory response begins to decrease from the third day after implantation [22]. Thus, it is essential to create an optimal microenvironment around implants to promote osseointegration by switching from the inflammatory phase to the regenerative phase at the appropriate time.

Meanwhile, osteoclasts are another important cell type involved in osseointegration because a successful bone healing/regeneration process relies on a fine-tuned reconstruction of various tissue components, which requires a delicate balance between bone formation mediated by osteoblast, and bone resorption mediated by osteoclast, to achieve bone homeostasis [23]. After implantation, monocyte/macrophage lineage cells firstly reach the surgical site [21], and are then differentiated into osteoclasts under the stimulation of endogenous receptor-activating factor nuclear factor-κ B ligand (RANKL) and macrophage colony stimulating factor (M-CSF) [24]. Afterwards, osteoclasts begin to initiate bone resorption around the implant by releasing protons and enzymes along with bleeding and inflammation [25]. Once the activity of osteoclasts exceeds that of osteoblasts, osseointegration of the implant will be adversely affected [26]. Therefore, efforts should be made to create a satisfied osteoimmunomodulatory microenvironment by subsiding inflammation and inhibiting osteoclast activity from the third day after implantation.

Interleukin-4 (IL-4) is a multifunctional cytokine, which is mainly expressed by activated T cells [27]. It can not only regulate immune cells to inhibit inflammation [28], but also inhibit osteoclast production in many ways [29,30]. Since IL-4 is easily inactivated and has a short half-life, it is essential to develop a drug-release system for its application [31]. Polydopamine (PDA) is a mussel-inspired polymer with good biodegradability and excellent biocompatibility [32,33]. Catechol and anthracene on its surface allow it to be modified by chemical reaction with thiols or amino compounds under alkaline conditions [34]. PDA has been used to immobilize IL-4 on titanium surfaces, though unfortunately it follows a monotonous release curve of IL-4 [35,36], which does not meet the demand of being released at specific time points to facilitate osteoimmunoregulatory function.

In order to obtain a programmed drug release, researchers have developed a number of drug delivery systems that could induce a controllable release of bioactive components by pH, ultrasound or near infrared (NIR) laser [37]. Among them, the photothermal-controllable release method attracted much attention owing to its potential ability to achieve precise drug release [38,39]. It has been reported that the photothermal effect after NIR laser irradiation could trigger functional protein release in mesoporous polydopamine with a built-in plasma nanoparticle core [40] and pesticide release in a composite structure with PDA as the core and poly (Nisopropylacrylamide) polymer as the shell, abbreviated as PDA@PNIPAm [41]. To realize the favorable effects of controllable release, the photothermal performance of TiO_2_ base material is also of great importance. It has been revealed that TiO_2_ anatase can effectively separate photoexcited charge carriers, which play a key role in photocatalytic application [42]. However, due to the wide band gap (≈3.2 eV) of TiO_2_ anatase, its light utilization rate is low [43]. Fortunately, it has been found that self-doping oxygen vacancy (OV) and Ti^3+^ defects into TNT by the electrochemical reduction method can yield black TiO_2_ nanotubes (B-TNT), whose light response range can be extended to visible light and even the NIR light area [43,44]. When irradiated by an NIR laser, B-TNT exhibits satisfactory photothermal performance [45]. 

The present study aimed to establish an ideal local immune microenvironment to suppress inflammatory reactions post-implantation and promote osseointegration by a photothermal-controlled release of IL-4 during the early stages after implant surgery. Herein, high-throughput DNA microarray detection followed by Gene Ontology (GO) enrichment analyses were employed to explore the potential osteoclast-related mechanism in macrophages after IL-4 addition. Then, a PDA/IL-4 photothermal-controlled release coating were superimposed onto the B-TNT surface to construct the B-TNT/PDA/IL-4 substrate. Afterwards, animal experiments were carried out to evaluate the in vivo effects of osseointegration. On 3 and 7 days after implantation, Hematoxylin-eosin (H&E), tartrate-resistant acid phosphatase (TRAP), inducible nitric oxide synthase (iNOS) and CD163 staining were used to observe inflammatory, TRAP^+^, iNOS^+^ and CD163^+^ cells. At 28 days post-implantation, micro computed tomography (micro-CT), H&E and Masson-trichrome (Masson) staining were used to evaluate new bone formation. We hypothesize that the established B-TNT/PDA/IL-4 surface could achieve accurate photothermal-controlled release of IL-4 on day 3 to create a favorable osteoimmunoregulatory microenvironment and inhibit osteoclast activity, which subsequently can promote early osseointegration at the bone-implant interface.

## 2. Materials and Methods

### 2.1. RNA Extraction, DNA Microarray, GO Enrichment Analysis

Firstly, we screened the differentially expressed genes in macrophages induced by RANKL after IL-4 application at 48 h. To screen the differential gene expression in macrophages after adding IL-4 and analyze the enrichment of the differential genes, RAW 264.7 cells (American Type Culture Collection, ATCC, Manassas, VA, USA) were cultured in a culture medium containing DMEM (Solarbio, Beijing, China), 10% fetal bovine serum (LIFE iLAB BIO, Shanghai, China), 1% penicillin/streptomycin (Invitrogen, Carlsbad, CA, USA) and 50 ng/mL mouse recombinant receptor activator of nuclear factor kappa-B ligand (RANKL, novoprotein, Shanghai, China) in an incubator at 37 °C in a 5% CO_2_ atmosphere. In the experimental group, IL-4 (Sino Biological, Beijing, China) was added to the culture medium at 48 h, and the final concentration was 200 ng/mL. After 4 days of cell culture, the total RNA was extracted by Trizol (Thermo Fisher Scientific, Waltham, MA, USA). Then, the RNA concentration and purity were determined by a Nanodrop spectrophotometer (NanoDrop Technologies, Wilmington, DE, USA).

Microarray analysis was performed using an Affymetrix Clariom S Assay microarray (Affymetrix, Santa Clara, CA, USA). The RNA was prepared and hybridized according to the Affymetrix user manual, and the data were assessed using the Robust Multichip Analysis (RMA) algorithm. Genes with multiple changes >1.5 or <−1.5 and *p* value < 0.05 were defined as DEGs (differentially expressed genes). Then, GO enrichment analysis was performed to analyze the different gene expressions according to gene ontology, and topGO’s combined *p* value was used to find overlap between the DE list and the GO annotation list. 

### 2.2. Fabrication of B-TNT

Herein, the electrochemical reduction method was used to prepare the B-TNT surface. Ti disks with a diameter of 14 mm and a thickness of 1.2 mm (ASTM F67 unalloyed Ti, grade 2; purity 99.7%; impurities content: O, 0.14%; Fe, 0.09%; C, 0.04%; N, 0.02%; other elements, 0.01%) were purchased from Baoji Titanium Industry (Baoji, Shanxi, China). Ti disks were polished by 400, 600, 800 and 1200 grit sandpaper in sequence, and then ultrasonically washed with acetone, ethanol and deionized water for 10 min in turn, dried in air and named Ti. Then, the TiO_2_ nanotube structure was fabricated by electrochemical anodization. In brief, Ti and a platinum sheet were connected to an anode and cathode, respectively, and immersed in 0.3 wt% ammonium fluoride ethylene glycol (NH_4_F, Aladdin, Shanghai, China; ethylene glycol, Tianjin Bohua Chemical Reagent Co., Ltd., Tianjin, China) electrolyte containing 2% deionized water by volume. Ti was manufactured under a constant voltage of 40 V for 1.5 h by a high-voltage DC power supply (Dongwen High Voltage Power Supply Factory, Tianjin, China) and then ultrasonically rinsed in ethanol for 15 min, dried by nitrogen (N_2_), annealed at 450 °C for 3 h, and named as TNT surface. 

Afterwards, the cathodic reduction method was used to fabricate black TiO_2_ nanotube surfaces with good photothermal properties. TNT and platinum discs were used as the cathode and anode, respectively, and soaked in 0.3 wt% NH_4_F ethylene glycol solution with a constant voltage of 40 V for 200 s. Then, the samples were ultrasonically rinsed in ethanol for 5 min, dried by N_2_, heat-treated at 45 °C for 3 h under N_2_ atmosphere, and denoted as B-TNT.

### 2.3. Fabrication of B-TNT/PDA/IL-4

PDA and IL-4 were loaded on B-TNT by the soaking method. To conjugate the PDA coating to the B-TNT, the B-TNT samples was immersed in 1 mg/mL solution of dopamine (10 mM Tris buffer, pH = 8.5) for 24 h in the dark and washed with deionized water three times. The acquired sample was labeled as B-TNT/PDA. Then, B-TNT/PDA was immersed in 400 ng/mL IL-4 solution for 24 h at 4 °C, washed with sterile phosphate-buffered saline (PBS) three times, and defined as B-TNT/PDA/IL-4 substrate.

### 2.4. Surface Characterization

After the preparation of the material, the surface morphology was observed, and the surface chemical composition was analyzed. A field-emission scanning electron microscope (FESEM, JEOL JSM-7100F, Tokyo, Japan) was applied to analyze the surface morphology of the samples. Image J software (ver. 1.53, National Institutes of Health, Bethesda, MD, USA) was used to quantitatively analyze the diameter and wall thickness of the nanotube structures in SEM images. Each group randomly measured the thickness of 5 nanotubes. One-way ANOVA followed by Tukey’s post hoc test was carried out to determine the statistical significance between the various samples. X-ray photoelectron spectroscopy (XPS; Axis Ultra DLD; Kratos Analytical; Manchester, UK) was used to determine the surface element composition and chemical properties of the samples. 

### 2.5. Photothermal Performance

The photothermal performance of Ti, B-TNT/PDA and B-TNT/PDA/IL-4 were examined. Samples were placed in 24-well plates with 1 mL/well PBS and then irradiated with an NIR laser (Hi-Tech Optoelectronics Co., Ltd., Beijing, China) at 808 nm and 0.4 W/cm^2^ for 5 min. A thermal imaging infrared camera, Flir E50 (Wilsonville, OR, USA), was used to measure the temperature every 30 s. 

### 2.6. Drug Release Test

Next, the release of IL-4 from the surface of B-TNT/PDA/IL-4 with or without an 808 nm NIR laser was tested. To detect the release of IL-4 from the surface of B-TNT/PDA/IL-4 with or without an 808 nm NIR laser, different samples were put into wells of a 24-well plate, one sample for one well, and then samples were incubated with 1 mL PBS for 3 days at 37 °C. At 48 h, the B-TNT/PDA/IL-4+Laser groups were irradiated with an NIR laser at 808 nm and 0.4 W/cm^2^ for 15 min, while the B-TNT/PDA/IL-4 group was left untreated. At each time point, the supernatant was completely collected for analysis and 1 mL of fresh PBS was added to every well to replace the removed solution. The IL-4 release from in each sample was determined by an IL-4 ELISA kit (Meike, Suzhou, China) according to manufacturer’s instruction.

### 2.7. In Vivo Experiments

#### 2.7.1. Surgical Procedure

Animal experiments were used to explore the effect of various materials on promoting osseointegration. The animal experiments in the present study were approved by the Animal Ethics Welfare Committee (AEWC) of Tianjin Hospital of Itcwm Nankai Hospital (approval no. NKYY-DWLL-2020-147). Twenty-seven 8-week-old male Sprague−Dawley (SD) rats were randomly divided into three groups (Ti, B-TNT/PDA/IL-4, B-TNT/PDA/IL-4+Laser), n = 3 for each group at each time point (3, 7, 28 days post-implantation). The animals were anesthetized by administering sodium pentobarbital (50 mg/kg body weight) intraperitoneally, with supplemental local anesthesia obtained using 2% lidocaine with epinephrine (1:100,000). Ti implants were purchased from Baoji Titanium Industry (ASTM F67 unalloyed Ti grade 2, Baoji, China), and fabricated into screwed cylindrical implants (diameter of 1.5 mm and highness of 4.5 mm). Then, the right hind limb of rats was shaved and disinfected. After the joint and joint capsule were cut and the tibial plateau was exposed, a channel from the tibial plateau to the medullary cavity was prepared using a power drill (Φ = 1.5 mm). After different samples were implanted, the muscle and skin were cleaned and sutured, respectively. At 48 h after implantation, the implant surface of B-TNT/PDA/IL-4+Laser groups were irradiated with a laser (808 nm, 0.4 W/cm^2^) for 15 min. Then, after surgery, the animals were sacrificed on the 3rd, 7th and 28th day via overdose injection of sodium pentobarbital and the tibial tissue with implants were removed and preserved for subsequent experiment.

#### 2.7.2. H&E, iNOS, CD163 and TRAP Staining on 3- and 7-Days Post-Implantation

Inflammatory cell, TRAP, iNOS and CD163 positive cell expression were observed using tissue section staining at the early stage post-implantation. All harvested samples were soaked in 17% EDTA decalcification fluid (Servicebio, Wuhan, China) for decalcification, and dehydrated with gradient ethanol. Then, the samples were embedded in paraffin and cut into 4μm slices to prepare the sections for later use. H&E staining, and immunohistochemical staining of TRAP, iNOS and CD163 were performed for samples at 3- and 7-days after surgery. Finally, the stained sections were observed, and images were taken by an optical microscope with a digital camera (Nikon ECLIPSE 90i, Tokyo, Japan). Quantitative analysis of the inflammatory cells, TRAP^+^, iNOS^+^ and CD163^+^ cells was conducted to compare the inflammatory status, prevalence of osteoclast, M1 and M2 macrophage cells between the different groups using the Image J software (ver. 1.53, National Institutes of Health, Bethesda, MD, USA) mentioned above.

#### 2.7.3. Micro-CT Analysis, H&E and Masson Staining on day 28 Post-Implantation

Micro-CT and H&E and Masson stainings were used to observe the bone formation around the implant. Different samples were scanned using a micro-CT system (Skyscan 1276, Bruker, Kontich, Belgium). In addition, the 3D images were reconstructed by the Nrecon software from the Skyscan Company (version 1.6, Kontich, Belgium). Quantitative analysis of bone volume/total volume (BV/TV), trabecular number (Tb.N), trabecular thickness (Tb.Th) and trabecular separation (Tb.Sp) were carried out by the CTAn program (ver. 1.17, Skyscan Company, Kontich, Belgium). Sections taken on day 28 post-implantation were further assessed by H&E and Masson staining. In addition, semi-quantitative analysis of new bone formation was carried out based on Masson staining sections using the above-mentioned Image J software.

### 2.8. Statistical Analysis

One-way ANOVA followed by Tukey’s post hoc test was carried out to determine the statistical significance between the various samples. Data were expressed as mean ± standard deviation (SD) of the three independent experiments. Values of *p* < 0.05 were regarded as statistically significant. 

## 3. Results

### 3.1. DNA Microarray Analysis

**Figure 1:**
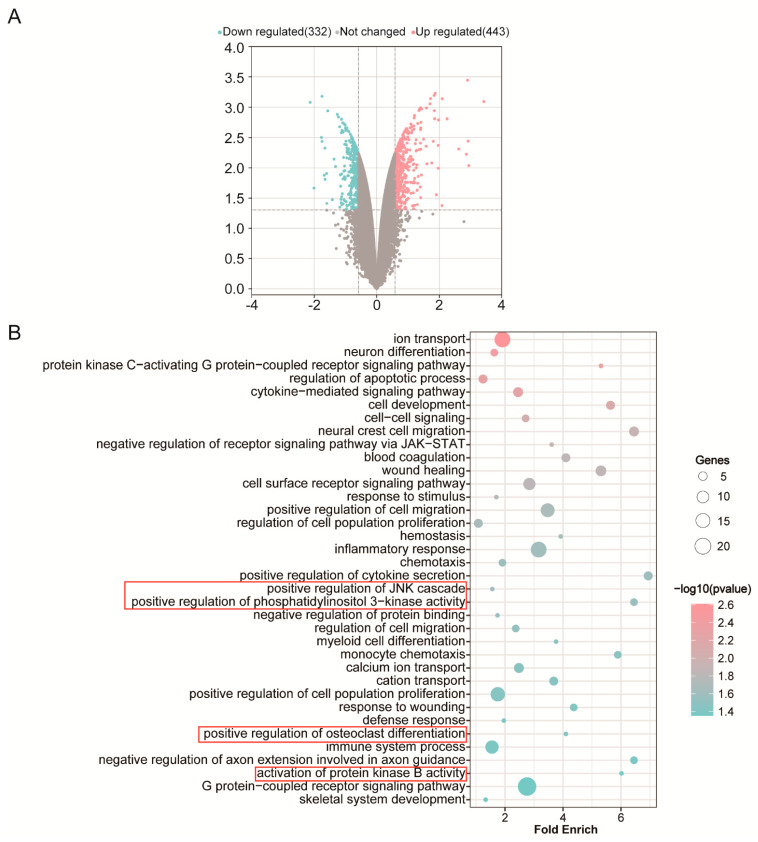
(**A**) Volcano plots showing the identified up-regulated (pink dots) and down-regulated (green dots) genes by IL-4 application. (**B**) Downregulated Gene Ontology (GO) terms in biological processes by IL-4 addition.

Using the microarray analysis technique, we firstly screened the differential gene expression profile in macrophages after adding IL-4. The Volcano map showed that 775 different genes, including 332 down-regulated genes and 443 up-regulated genes, were identified as being affected by IL-4 addition (Figure 1A). In this experiment, we focused on the effect of IL-4 on osteoclast differentiation induced by RANKL. 

**Figure 2:**
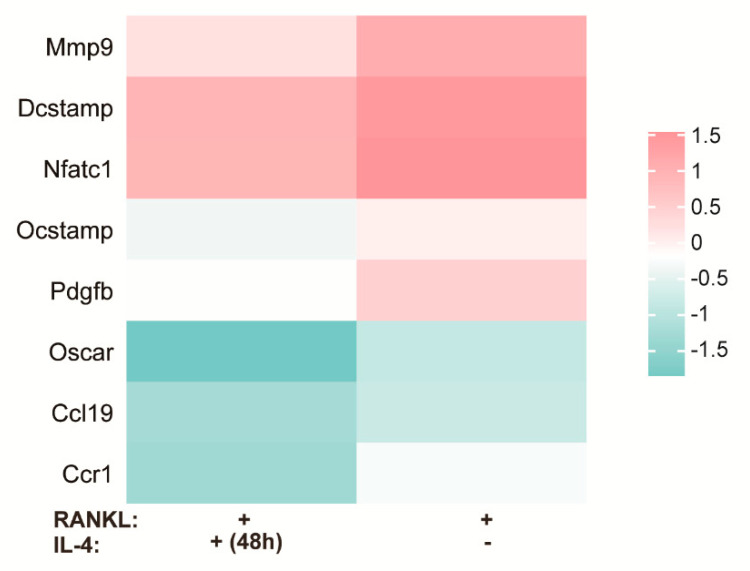
Down-regulated osteoclast differentiation related genes after IL-4 treatment.

Previously, several signal pathways have been reported to play a role in osteoclast differentiation. After RANKL binds to its receptor RANK, the PI3K/AKT and MAPK (such as ERK, JNK and P38) signaling pathways were activated, and then osteoclasts are formed [46]. Our results revealed the down-regulated GO terms in biological processes included positive regulation of JNK cascade, positive regulation of phosphatidylinositol 3−kinase activity, positive regulation of osteoclast differentiation and activation of protein kinase B activity (Figure 1B). Additionally, Figure 2 shows that genes down-regulated by IL-4 consisted of Nfatc1, Mmp9, Oscar and Dcstamp, which are closely related to osteoclast production. Collectively, these results indicate that the addition of IL-4 at 48 h inhibited osteoclast differentiation and function induced by RANKL. 

### 3.2. Surface Characterization and Drug Release Behavior Detection

#### 3.2.1. Surface Morphology Characterization

**Figure 3:**
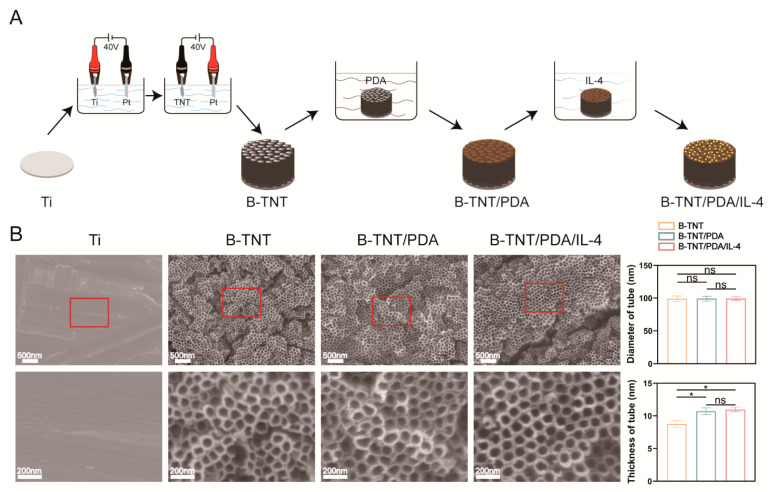
(**A**) Schematic illustration of the fabrication of different surfaces. (**B**) Representative scanning electron microscope (SEM) images and quantitative analyses of B-TNT, B-TNT/PDA and B-TNT/PDA/IL-4 surfaces. * indicates statistical difference between groups (*p* < 0.05); ns means no statistical difference between groups.

Figure 3A displays the schematic graph of the fabrication of different samples. As shown in Figure 3B, there were slight scratches on the surface of Ti formed by the polishing step, while after anodic oxidation and cathodic reduction, uniform nanoscale tubular structure was obtained on the B-TNT surface. After PDA and PDA/IL-4 coating, the nanotubular structure was still visible on both the B-TNT/PDA and B-TNT/PDA/IL-4 substrates. According to the quantitative analyses in Figure 3B, the mean diameters of surface nanotubes in B-TNT, B-TNT/PDA and B-TNT/PDA/IL-4 groups were 99.1 ± 4.2 nm, 99.1 ± 3.7 nm and 99.2 ± 3.1 nm, respectively, without statistical difference. Moreover, the average wall thickness of the B-TNT/PDA and B-TNT/PDA/IL-4 groups were 10.7 ± 0.5 nm and 11.0 ± 0.4 nm, respectively, which was higher than that of the B-TNT group (8.8 ± 0.5 nm). These results suggest that the nanotube structures were partially covered by the PDA and PDA/IL-4 coating.

#### 3.2.2. Surface Chemical Composition Characterization

**Figure 4:**
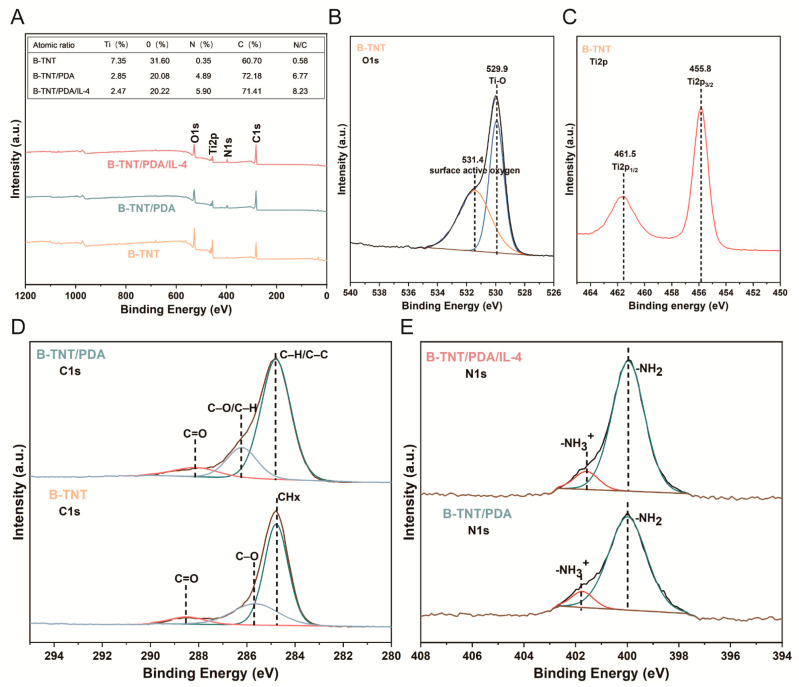
(**A**) Quantification of surface element composition and X-ray photoelectron spectroscopy (XPS) full-scan spectra of B-TNT, B-TNT/PDA and B-TNT/PDA/IL-4 surfaces. (**B**) XPS narrow-scan spectra of O1s for B-TNT surface. (**C**) XPS narrow-scan spectra of Ti2p for B-TNT surface. (**D**) XPS narrow-scan spectra of C1s for B-TNT and B-TNT/PDA surfaces. (**E**) XPS narrow-scan spectra of N1s for B-TNT/PDA and B-TNT/PDA/IL-4 surfaces.

XPS was used to further analyze the chemical composition of the material surface. The O1s peak came from B-TNT, while the C1s peak was partly due to environmental contamination. In addition, a little amount of N element was detected in the B-TNT group, which was probably due to the residual NH_4_F electrolyte. In Figure 4B, the narrow scanning spectrum of O1s element on the surface of B-TNT group exhibited two peaks at 529.9 eV and 531.4 eV, which can be ascribed to the Ti-O bond and surface active oxygen (chemisorbed by oxygen defects, e.g., oxygen vacancy) [47], respectively. It was reported that Ti 2p_3/2_ and Ti 2p_1/2_ peaks of Ti4^+^ are at 458.4 and 464.1 eV in TiO_2_, respectively [48]. In the Ti2p narrow scan spectrum of the B-TNT group in Figure 4C, it can be seen that Ti2p_3/2_ and Ti2p_1/2_ peaks were located at 455.8 and 461.5 eV, respectively. Consistent with previous studies, the peaks of Ti2p_3/2_ and Ti2p_1/2_ shifted to a lower binding energy, compared with Ti4^+^ [49]. This phenomenon can be attributed to the existence of Ti3^+^ in the B-TNT group. After PDA was attached to the surface of B-TNT, the obvious N1s peak on the B-TNT/PDA and B-TNT/PDA/IL-4 surfaces originated from the amino group of the dopamine molecule (Figure 4A). The narrow scanning spectrum of Figure 4E showed that most of the N elements on the surface of B-TNT/PDA existed in the form of amino groups and a few existed in the form of protonated amino groups. As shown in Figure 4D, there were three peaks in C1s of B-TNT, corresponding to CH_X_, C-O and C=O bonds [50]. The C1s of B-TNT/PDA also has three peaks, corresponding to C-H/C-C, C-O/C-H and C=O bonds [51]. Consistent with previous studies, our results also detected the same C1s bonds. Moreover, the peak area and peak intensity of the B-TNT/PDA group significantly increased compared to the B-TNT group. Since the loading of PDA may increase the content of C element (Figure 4A), these results further confirmed the successful loading of the PDA coating. Figure 4A reveals that the surface N content of the B-TNT/PDA/IL-4 group (5.90%) was higher than that of the B-TNT/PDA group (4.89%). Furthermore, the N/C ratio increased in the order of B-TNT, B-TNT/PDA and B-TNT/PDA/IL-4 groups. In addition, since IL-4 has a higher N content than that of the PDA molecule, the higher peak area of N1s of the B-TNT/PDA/IL-4 group than that of the B-TNT/PDA group implied the successful loading of IL-4. 

#### 3.2.3. Photothermal Performance and Drug Release Profile

**Figure 5:**
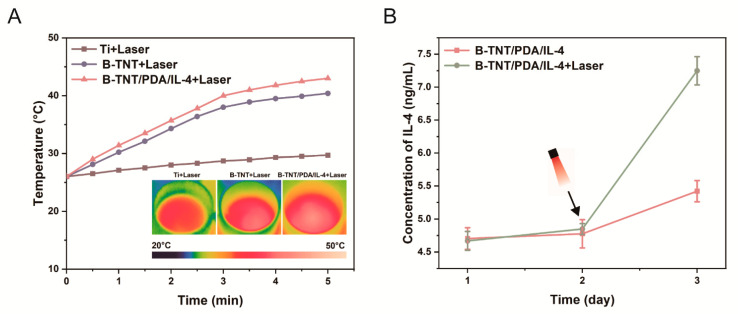
(**A**) Temperature changes in different surfaces irradiated by laser. (**B**) The amount of IL-4 released in B-TNT/PDA/IL-4 substrates with or without laser irradiation.

Figure 5A reveals the photothermal performance of different surfaces. Compared with Ti, the B-TNT and B-TNT/PDA/IL-4 substrates showed obvious photoinduced heating effects under near-infrared laser irradiation. Among them, B-TNT/PDA/IL-4 had the best photothermal effect, with the temperature rising to about 42 °C. 

Figure 5B displays that for the B-TNT/PDA/IL-4 without-laser group, the IL-4 concentration in the B-TNT/PDA/IL-4 surface was 4.70 ± 0.16 ng/mL and 4.78 ± 0.22 ng/mL on day 1 and day 2, respectively, and then increased slightly to 5.42 ± 0.16 ng/mL on day 3. For the B-TNT/PDA/IL-4+Laser group, the IL-4 concentration was 4.67 ± 0.14 ng/mL and 4.85 ± 0.08 ng/mL on day 1 and day 2, respectively, similar to that of the B-TNT/PDA/IL-4 group. After NIR irradiation at 48 h, the IL-4 concentration increased to 7.25 ± 0.22 ng/mL on day 3, which was 1.83 ng/mL higher than that of the B-TNT/PDA/IL-4 group, implying this achieved the on-demand controlled release of IL-4, as expected. 

### 3.3. In Vivo Effect Evaluation

**Figure 6:**
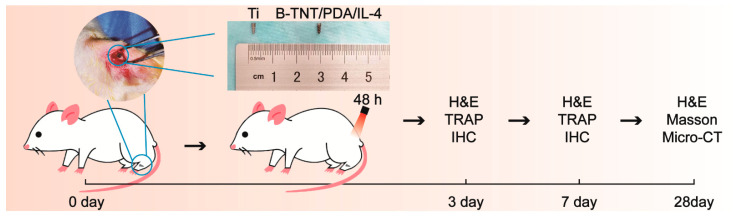
Schematic diagram of experimental design and implants used in the experiment.

Figure 6 shows the schematic graph of the animal experiments. See the Materials and Methods section for detailed experimental procedures. 

**Figure 7:**
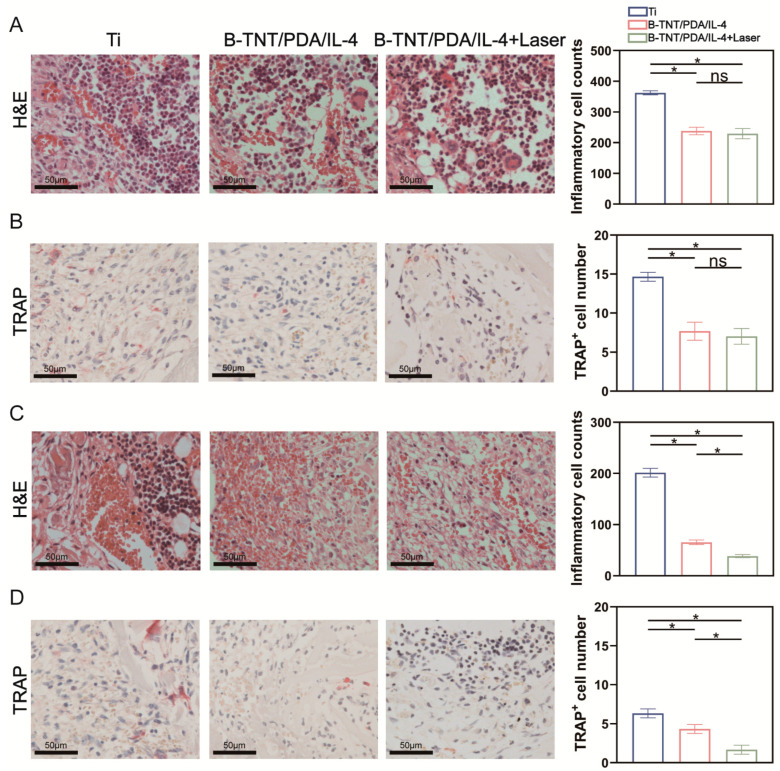
(**A**) Hematoxylin-eosin (H&E) staining images and quantitative assessment of inflammatory cells after 3 days of implantation. (**B**) Tartrate-resistant acid phosphatase (TRAP) staining images and quantitative assessment of TRAP^+^ cells after 3 days of implantation. (**C**) H&E staining images and quantitative assessment of inflammatory cells after 7 days of implantation. (**D**) TRAP staining images and quantitative assessment of TRAP^+^ cells after 7 days of implantation. * indicates statistical difference between groups (*p* < 0.05); ns means no statistical difference between groups.

Three days after implantation, H&E staining of the peri-implant tissues and quantitative analysis (Figure 7A) showed that the inflammatory cell infiltration of B-TNT/PDA/IL-4 and B-TNT/PDA/IL-4+Laser groups was basically the same, and less than that of the Ti group, *p* < 0.05. We also explored the effects of different materials on osteoclast production. TRAP staining and related semi-quantitative analysis showed that the number of osteoclasts in the Ti group were more than those in the B-TNT/PDA/IL-4 groups with or without laser, although no difference was found between the B-TNT/PDA/IL-4 and B-TNT/PDA/IL-4+Laser groups (Figure 7B). However, 7 days after implant surgery, the B-TNT/PDA/IL-4+Laser group had the least inflammatory cells and TRAP positive cells (Figure 7C,D). The B-TNT/PDA/IL-4+Laser group inhibited the inflammatory reaction and osteoclast differentiation in the early stage of implant implantation.

**Figure 8:**
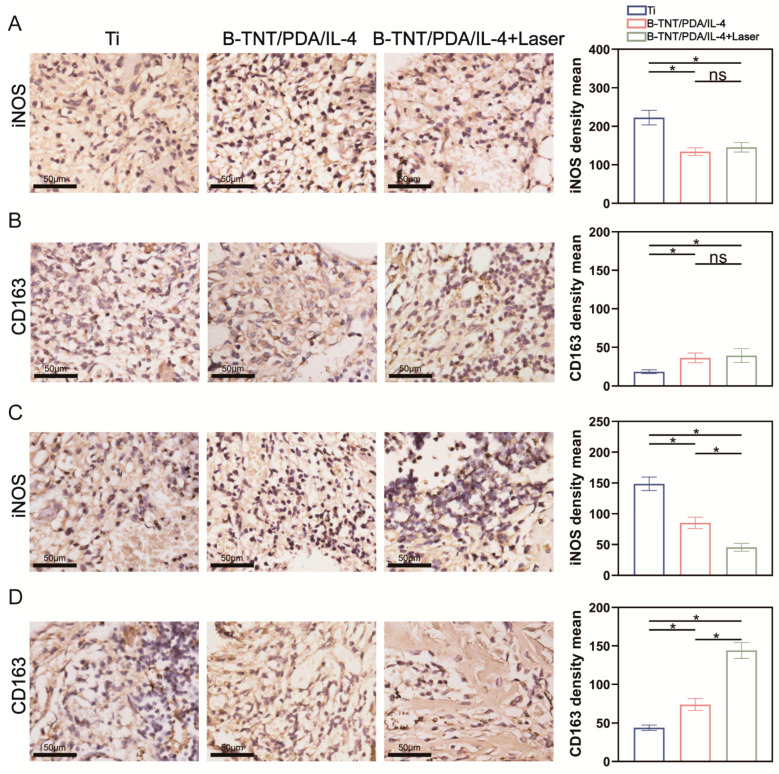
(**A**) Inducible nitric oxide synthase (iNOS) staining images and quantitative assessment of iNOS^+^ cells after 3 days of implantation. (**B**) CD163 staining images and quantitative assessment of CD163^+^ cells after 3 days of implantation. (**C**) iNOS staining images and quantitative assessment of iNOS^+^ cells after 7 days of implantation. (**D**) CD163 staining images and quantitative assessment of CD163^+^ cells after 7 days of implantation. * indicates statistical difference between groups (*p* < 0.05); ns means no statistical difference between groups.

Immunohistochemical (IHC) staining was also used to evaluate the immune environment around the implant. As shown in Figure 8A, the number of iNOS positive cells in the Ti group was higher than that of the B-TNT/PDA/IL-4 and B-TNT/PDA/IL-4+Laser groups. On the contrary, the number of CD163 positive cells in the Ti group was lower than that of the B-TNT/PDA/IL-4 and B-TNT/PDA/IL-4+Laser groups 3 days after implantation (Figure 8B). At 7 days after implantation, iNOS positive cells in each group showed a decreasing trend, while CD163 positive cells revealed an increasing tendency (Figure 8C,D) compared with those at 3 days after implantation (Figure 8A,B). These results suggest that 7 days after implantation, the immune inflammation response relieved in all groups. It is also worth noting that the B-TNT/PDA/IL-4+Laser group had the least iNOS positive cells, and the most CD163 positive cells after 7 days of implantation, suggesting it has the best effects for shifting the immune-microenvironment from an M1 inflammatory status to an M2 restorative status, so as to facilitate osteogenesis.

**Figure 9:**
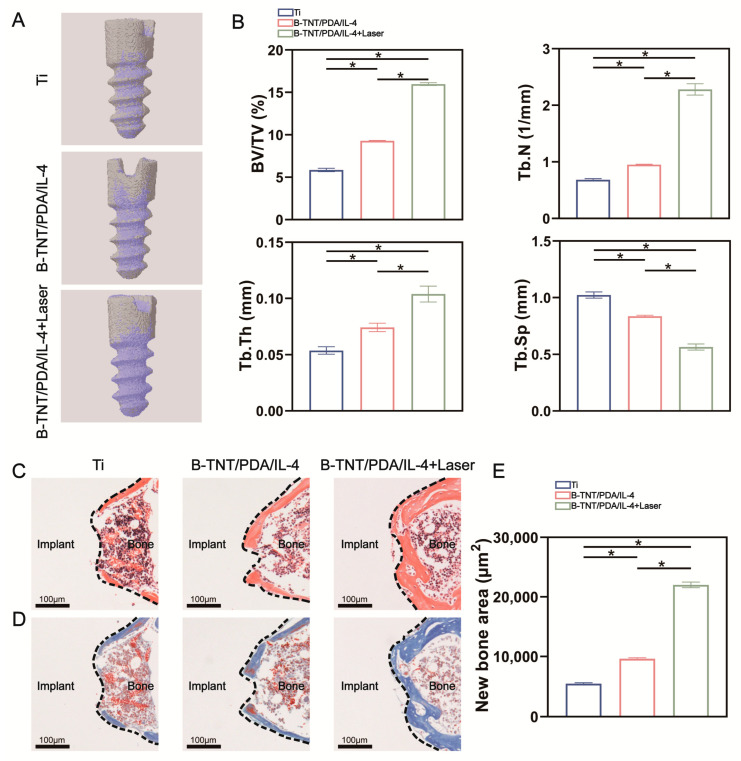
(**A**) Micro-CT 3D reconstruction images of different samples, with gray color representing implants, while purple color representing new bone formation. (**B**) Quantitative analysis of bone volume/total volume (BV/TV), trabecular number (Tb.N), trabecular thickness (Tb.Th) and trabecular separation (Tb.Sp) values. (**C**) H&E and (**D**) Masson staining images of different samples 28 days after implantation. (**E**) Quantitative analysis of new bone formation area. * indicates statistical difference between groups (*p* < 0.05).

Figure 9A demonstrates the reconstructed 3D images 4 weeks after implantation, with gray and purple colors representing implant and new bone formation, respectively. It was seen that the largest amount of new bone formation around the implant were observed in the B-TNT/PDA/IL-4+Laser group, followed by the B-TNT/PDA/IL-4 and Ti groups. The quantitative analyses showed the same tendency. The B-TNT/PDA/IL-4+Laser group had the highest BV/TV, Tb.Th and Tb.N values and the lowest Tb.Sp values (Figure 9B). Subsequently, H&E and Masson staining and semi-quantitative analysis were also used to evaluate new bone formation. The results reveal that less osteoid tissues were observed in the Ti and B-TNT/PDA/IL-4 groups, while a large amount of new osteoid tissue was observed in the B-TNT/PDA/IL-4+Laser group, indicating its superior osteogenic ability (Figure 9C–E). 

In vivo experimental results reflected that in the early stage of implantation, the release of IL-4 on the surface of B-TNT/PDA/IL-4 was controlled by NIR 808 nm laser, which effectively promoted early osseointegration of implants by timely relieving tissue inflammation, inhibiting osteoclast production and creating a pro-osteogenic immune microenvironment.

## 4. Discussion

Previously, numerous strategies have endeavored to modify dental implant surfaces to promote osteogenesis in osteoblast cells and mesenchymal stem cells and have acquired satisfactory effects to some extent. However, in order to achieve early osseointegration in adverse systematic conditions, including osteoporosis, uncontrolled DM and cancer, etc., solely relying on osteogenesis-related cells, such as osteoblast and mesenchymal stem cell (MSC), to improve bone formation is not enough. The reason lies in that after implant surgery caused tissue injury, would healing occurs, comprising 4 stages: hemostatic, inflammatory, proliferative and remodeling stages. The timely shift from the inflammatory stage to the proliferative phase plays an important role in successful wound healing, and failed transition leads to compromised implant osseointegration [52]. During this transition, immune cells including macrophages and neutrophils orchestrate an effective healing process through changing their phenotype and recruiting proliferative phase cells to the peripheral area of implant [53]. In addition to the well-known effects on inflammation, immune cells also release cytokines to regulate osteogenesis, thus inhibiting or inducing bone formation.

Among all kinds of immune cells, macrophages are often regarded as the excellent candidates for bone immunomodulation because they not only serve as critical inflammation modulators but also have dynamic crosstalk with bone cells and are crucial for normal bone healing and formation [54]. Moreover, macrophage lineage is also one of the sources of osteoclasts [55]. Previous studies have shown that osteoclasts can secrete active transforming growth factor-β1 (TGF-β1) and platelet-derived growth factor-BB (PDGF-BB) to induce bone formation and angiogenesis [25]. However, once the osteoclast activity is too high, it will cause excessive bone absorption, which is unfavorable to bone integration [56]. Therefore, the immune system serves as a double-edged sword in regulating osseointegration [16]. In the present study, we hoped to employ a surface modification strategy to create a favorable immune microenvironment around implants to improve osteogenesis. 

It has been reported that 72 h of inflammatory status is necessary for normal osteogenesis and inflammation and osteoclast activity should decline to ensure positive regulation of osteogenic differentiation from the third day after implantation [54]. Hence, supplementing external factors at an appropriate time is an effective way to accurately control the immune response to achieve osteoimmunomodulation. IL-4 release on the surface of biomaterials has been proven to improve osteoimmunomodulation by regulating the macrophage phenotype [57]. However, few studies have reported IL-4 promoting osteogenesis by simultaneously inhibiting inflammation and osteoclast activity.

Herein, we first screened the differentially expressed genes in macrophages after IL-4 application at 48 h. The osteoclast forming process involves the proliferation, migration, cell–cell adhesion and fusion of osteoclast precursor, and finally forms mature multinucleated osteoclasts [58]. In this process, M-CSF and RANKL are the two key cytokines. M-CSF mainly stimulates migration and promotes osteoclast precursor proliferation [58], while RANKL binds to its receptor, RANK, and drives downstream signals, leading to osteoclast differentiation [59]. Both RANKL and M-CSF could be added to culture media to induce osteoclast production [25,60]. In the present experiment, since we focused on the effect of IL-4 on osteoclast differentiation, RANKL was added to the medium for inducing osteoclast differentiation. Song et al. [61] showed that a RANKL concentration of 30–100 ng/mL could independently induce osteoclast formation. Both Kwang et al. [62] and Nadia et al. [55] added 50 ng/mL RANKL to the culture medium to successfully induce osteoclast formation. Therefore, we chose a moderate concentration of RANKL (50 ng/mL) which successfully induced osteoclast formation in our preliminary experiment. IL-4 could reversibly arrest RANKL-mediated osteoclast formation by blocking JNK, p38 and ERK mitogen-activated protein kinase pathways [63]. It has been suggested that the PI3K/AKT [46] signaling pathway is involved in osteoclast formation. Our GO-term enrichment analysis results were consistent with previous reports. Moreover, IL-4 could suppress osteoclast formation by inhibiting NFATc1 expression induced by RANKL [64]. Mmp9, Oscar, Ocstamp and Dcstamp are key osteoclast differentiation-related genes [65,66]. Activation of CCR1 [67] and Pdgfb [68] increases osteoclast formation, while CCL19 [69] plays an important role in bone destruction by increasing osteoclast migration and absorption activity. The down-regulated NFATc1, Mmp9, Oscar, Ocstamp, Dcstamp, Pdgfb, Ccl19 and Ccr1 gene levels by IL-4 further illustrated the inhibitory effect of IL-4 on osteoclast differentiation. 

Therefore, in the current study, we loaded IL-4 on the B-TNT surface by PDA to fabricate the B-TNT/PDA/IL-4 substrate. Then, the accurate release of IL-4 on the third day after implantation was fulfilled by the photothermal-controlled release characteristics of the established B-TNT/PDA/IL-4 substrate under NIR laser irradiation. 

The research group has conducted many studies on the preparation of TNT. HF was used as the anode oxidation electrolyte initially, while an electrolyte composed of NH_4_F, ethylene glycol and water were used in this study. The reason is that the solvent has a high viscosity, which can reduce the diffusion rate of fluorine ions in the electrolyte to obtain controllable and orderly arranged nanotubes [70,71]. In addition, considering the toxicity and volatility of HF solution, an electrolyte composed of ammonium fluoride, ethylene glycol and water was used for the preparation of TNT. In addition, previous studies by the research group have shown that the diameter of nanotubes increases with the increase in voltage [12] and nanotubes with a diameter of 70–100 nm can induce cell adhesion and osteoblast differentiation [71]. Therefore, this study continued the 40 V anodic oxidation voltage used by the research group to prepare large-diameter (≈100 nm) nanotubes. As for the preparation of B-TNT, most of the reported literature uses the hydrogen reduction method in a high temperature environment [72,73], which has the disadvantage of complex and dangerous procedures. Ma et al. introduced the generation of oxygen vacancies on the surface of TNT by electrochemical cathodic reduction [44], which we employed to manufacture the B-TNT in this work. In a study, Zhu et al. prepared black titanium dioxide nanotube arrays by electrochemical reduction at a set voltage (30, 35, 40, 45 and 50 V) for a certain time (15, 20, 30, 60 and 120 min). The results show that extra high voltage and extra long time will destroy the pore structure of nanotubes [74]. In addition, Li et al. [75], Yu et al. [76] and Ma et al. [44] prepared black titanium dioxide nanotubes at 40 V for 200 s, 60 V for 30 s and 60 V for 35 s, respectively. Our preliminary experiment revealed that black TiO_2_ nanotubes with uniform tubular structures were successfully prepared at 40 V for 200 s. Therefore, we fabricated black nanotubes at 40 V for 200 s in the present experiment. The presence of Ti3^+^ and oxygen vacancies (Figure 4B, C) leads to the generation of black TiO_2_ nanotubes, enhances the light absorption in the visible and near-infrared regions and promotes photothermal conversion [45]. Ma et al. also showed that the electronic transition from oxygen vacancies and Ti3^+^ localized states to conduction bands and the electronic transition from valence bands to oxygen vacancies and Ti3^+^ localized states are responsible for the NIR laser absorption of B-TNT [44]. After loading PDA and IL-4, the diameter of the nanotubes remained unchanged, and the wall became thicker than that of the B-TNT group (Figure 3B). This indicates that PDA and IL-4 were distributed in the opening and the inner wall of the nanotube. 

The photothermal performance examination of different materials showed that the B-TNT/PDA/IL-4 group had the best photothermal effect (Figure 5A). The reason lies in that PDA is also a photothermal agent [77], which works synergistically with B-TNT on the B-TNT/PDA/IL-4 substrate. A Previous study has shown that 40~42 °C is beneficial to bone regeneration [78], which was the temperature reached by B-TNT/PDA/IL-4 after being irradiated by NIR laser in our experiment. 

Considering the drug delivery needs of different diseases, scientists have introduced “programmed” and “on-demand” drug delivery methods, which could control the release time and quantity of drugs to fulfil ideal treatment effects [79]. As shown in Figure 5B, NIR laser irradiation at 48 h successfully triggered a controlled on-demand release of IL-4 from the surface of B-TNT/PDA/IL-4 at day 3 post-implantation, which is the optimal timing for inflammation to subside during implant osseointegration [54]. 

Next, in vivo experiments were used to evaluate the effect of the established B-TNT/PDA/IL-4 substrate on osseointegration. We previously reported that icariin/aspirin composite coating on the TNT surface could induce immunoregulatory function of macrophages and improve osteoblast activity [71]. Moreover, Zn-incorporated TNT surfaces may transfer the macrophages from M1 to M2 phenotypes to create an osteogenic microenvironment and accelerate bone formation [12]. It has been well-accepted that macrophages are assumed to play a key role in modulating bone-implant osteogenesis [80]. Considering the important role osteoclasts played in maintaining the dynamic balance of bone homeostasis with osteoblasts, we intended to explore the effects of osteoclast on osseointegration in the present work. IL-4 is a classic M2 polarized cytokine [81], which could inhibit osteoclast differentiation [29]. Histological staining and quantitative analysis in Figure 7 and Figure 8 show that after three days of implantation, decreased inflammatory cell counts, TRAP^+^ cell number and iNOS^+^ cells and increased CD163^+^ cells were observed compared with the Ti group, although no difference was detected between the B-TNT/PDA and B-TNT/PDA/IL-4+Laser groups. These results implied that an IL-4 release could exert the immunoregulatory function of anti-inflammation and inhibit osteoclast differentiation to some extent, while it still took some time for the B-TNT/PDA/IL-4+Laser to exhibit an obvious positive immunoregulatory role. Seven days after implantation, the B-TNT/PDA/IL-4+Laser substrate displayed the lowest inflammatory, TRAP^+^, iNOS^+^ and highest CD163^+^ cells among the three groups, with statistical significance. These results revealed the superior osteoimmunoregulatory role of the B-TNT/PDA/IL-4+Laser substrate and its ability to inhibit osteoclast activity. There are many reports about the mechanism of IL-4 inhibiting osteoclast differentiation [30,63]. IL-4 can inhibit osteoclast differentiation by inhibiting RANKL expression in TNF-α-activated stromal cells [30], and block NF-κB activation and JNK, p38 and ERK mitogen-activated protein kinase pathways [63]. The mechanism of IL-4 inducing macrophages to differentiate into the M2 phenotype may be through up-regulating the expression of JNK and its downstream transcription factor c-Myc [82].

Although the specific regulatory mechanism of IL-4 on osteoclast differentiation remains to be studied, our results proved that under NIR laser irradiation, the photothermal-controlled B-TNT/PDA/IL-4+Laser substrate released a large amount of IL-4 on the third day after implantation, which successfully shifted the immune microenvironment from the inflammatory to the regenerative stage and inhibited osteoclast formation. Micro-CT and histological staining are commonly used to evaluate bone formation around biomaterials [26,83]. The Micro-CT and histological assessments in Figure 9 show that the B-TNT/PDA/IL-4+Laser group had superior osteogenesis ability than the other two groups. As we expected, the established B-TNT/PDA/IL-4 group efficiently released IL-4 on the third day after implantation in SD rats, so as to create an optimal immune microenvironment to promote the osseointegration of implants.

## 5. Conclusions

In this study, the established B-TNT/PDA/IL-4 substrate successfully achieved precisely controllable release of IL-4 on the third day after implantation. In vivo experiments further showed that the B-TNT/PDA/IL-4 group could reduce the inflammatory response, inhibit osteoclast differentiation and finally promote osseointegration of implants. Our work provides a new strategy to modify implant surfaces through modulating the pro-osteogenic immune microenvironment for improving osseointegration.

## Data Availability

Data are available on request.
